# Impact of early life famine exposure on adulthood anthropometry among survivors of the 1983–1985 Ethiopian Great famine: a historical cohort study

**DOI:** 10.1186/s12889-020-09982-x

**Published:** 2021-01-07

**Authors:** Getachew Arage, Tefera Belachew, Kemal Hajmahmud, Mubarek Abera, Fedilu Abdulhay, Misra Abdulahi, Kalkidan Hassen Abate

**Affiliations:** 1 Department of Nutrition and Dietetics, College of Health Sciences, Debre Tabor University, Debre Tabor, Ethiopia; 2 Department of Nutrition and Dietetics, Institute of Health, Jimma University, Jimma, Ethiopia; 3 Department of Psychiatry, Faculty of Medical Sciences, Jimma University, Jimma, Ethiopia; 4 Department of Obstetrics and Gynecology, Faculty of Medical Sciences, Jimma University, Jimma, Ethiopia; 5 Department of Population and Family Heath, Institute of Health, Jimma University, Jimma, Ethiopia

**Keywords:** Famine exposure, Anthropometric measurements, Early life, Ethiopian great famine

## Abstract

**Background:**

Nutritional insult in early life brings adaptive changes in body structure and functioning that could remain throughout the affected individual’s life course. The long term impact of early life famine exposure on adulthood anthropometric measurements has been recorded in previous studies. However, the results were contradictory. Hence, we extend this study to examine the impact of famine exposure during early life on adulthood’s anthropometry among survivors of the 1983–85 Ethiopian great famine.

**Methods:**

A total of 1384 adult men and women survived from 1983 to 85 Ethiopian great famine were included in the study. Famine exposure status was classified into five groups: early life-exposed, prenatal-exposed, postnatal-exposed, adolescence-exposed, and non-exposed based on self-reported age and birthdate of the participants. Prenatal, post-natal, and adolescence exposed groups were considered as early life exposed. Following a standard procedure, anthropometric measurements were taken. A linear regression analysis was used to analyze the impact of famine exposure on adult anthropometric measurements adjusted for all possible covariates. The effect of famine exposure on overweight, general obesity, and abdominal obesity was examined using multinomial and binary logistic regression analysis.

**Result:**

Compared to non-exposed groups, adult height was lower by 1.83 cm (β = − 1.83; 95% CI: − 3.05, − 0.58), 1.35 cm (β = − 1.35; 95% CI: − 2.56, − 0.14) and 2.07 cm (β = − 2.07 cm; 95% CI: − 3.31, − 0.80) among early life, prenatal and post-natal exposed groups, respectively. Likewise, famine exposure during early life (β = 0.02; 95% CI: 0.01, 0.03), prenatal (β = 0.03; 95% CI: 0.02, 0.03) and post-natal life (β = 0.02; 95% CI: 0.02, 0.03) was positively associated with increased waist to height ratio. However, none of the above exposures resulted in a significant association with body mass index (*P > 0. 05*). Additionally, exposure to famine during early stage of life was not associated with increased risk of overweight, general obesity and abdominal obesity in adults.

**Conclusion:**

Decreased adult height and increased waist-to-height ratio were associated with early life exposure to famine, particularly prenatal and post-natal exposure. These results therefore underscore the significance of avoiding undernutrition in early life, which tends to be important for achieving once potential adult height and to minimize the increased risk of anthropometric markers of abdominal obesity such as waist to height ratio in later life.

## Background

Anthropometric measurements include height, weight, waist circumference, hip circumference, body mass index (BMI), waist to hip circumference ratio (WHR) and waist to height ratio (WHtR) [1]. The Body Mass Index (BMI) is defined as the weight in kilograms divided by the height of the square in metres, which is widely used to describe overweight or obesity and is closely related to the degree of body fat in most settings [2]. Waist circumference, waist to hip circumference ratio (WHR) and waist to height ratio (WHtR) are anthropometric measures for abdominal obesity [3, 4]. These adult anthropometric indices are determined during prenatal (intrauterine), post-natal (first 2 years) and adolescence (10–19 years) periods of life[5, 6], which are crucial periods of human growth and development. Intrauterine life, first 2 years of post-natal life and adolescence periods are critical times where the body employs reductive adaptive mechanisms to sustain life at the expense of influencing the future adulthood for the worst [6–8].

Overweight, general obesity and abdominal obesity in adults are established risk factors for metabolic diseases, including diabetes mellitus, hypertension, cardiovascular illness, stroke, certain types of cancer, and respiratory diseases [1–3]. Studies also indicated that adult height is inversely associated with the risk of cardiovascular diseases [5] and positively associated with productivity [6]. Each added cm of adult height is associated with an almost 5% increase in wage rates [9].

Body composition measurements such as body mass index (BMI), waist circumference, waist to hip ratio (WHR), and waist to height ratio (WHtR) are affected by adult’s lifestyle factors such as unhealthy dietary habit, physical inactivity, cigarette smoking and alcohol drinking [10–12]. On the other hand, adult height is determined by the cumulative result of the interaction between genetics and environmental factors, including dietary, socio-economic, and health conditions in early life [11, 13, 14]. Recent evidence from studies focusing on secular trends of adult anthropometric measurements showed its intricate relation with nutrition in early life [15–18]. This phenomenon is well summarized by Barker and colleagues, who introduced the concept of “Fetal Origin of Adult Diseases (FOAD)” or “Developmental Origins of Health and Disease (DOHaD)” [19–21]. This theory indicated that nutritional insult in early life leads to permanent changes in body structure, physiology, and metabolism, thereby influencing adulthood diseases, including anthropometric measurements [22].

Famine can serve as a natural laboratory model to test the DOHaD hypothesis in humans, where undernutrition is considered as natural exposure [23]. More specifically, global famine such as the Ethiopian great famine of 1983–85 can be taken as natural exposure to explore the long-term assaults of early-life undernutrition. The Ethiopian great famine of 1983–1985 affected whole Ethiopia and returned to normal year (but some problems in certain villages) from September 1986 to September 1987. On the scale of famine, it was ranked “Great Famine” because of its global devastating effects on the country, compared to other famines that have ever existed in Ethiopia or Africa . Political factors in the country caused undue delay in relief interventions in the form of food aid [24].

Findings from an earlier investigation of famine in early life and anthropometric indices of adulthood have been documented in Dutch [25–29] and Chinese [30–33] birth cohorts; however, the findings were varied. There is lack of information on the long-term consequences of early-life exposure to famine in Ethiopia, a country with an unsettled situation with ongoing maternal and childhood malnourishment. Presumably, this study may explain the developmental basis of adulthood diseases beyond established risk factors. It would be of great interest to clarify the impact of childhood and maternal undernutrition during pregnancy in an African sample. Hence, this study could test the long-term impact of early life exposure to the 1983–85 Ethiopian great famine on adulthood anthropometric parameters among famine survivors in Wollo province, Ethiopia.

## Methods

### Study design and setting

From March 15 to April 30, 2019, a historical cohort study was conducted. Information on the research settings was published elsewhere [34]. This study was briefly carried out in North Wollo Zone, Raya Kobo district, which was the origin of the great famine in Ethiopia between 1983 and 1985 [24].

### Study participants

Adults exposed to the Ethiopian great famine during early life (prenatal, first 2 years of post-natal and adolescence period of life) were the study participants. Participants were divided into the following groups linked to the period of famine (August 1983 to August 1985 [24] a historical cohort study was: prenatal exposure (age = 34–36, birth year = 8 August 1983 to 30 August 1985), post-natal exposure (age = 37–38, birth year = 8 September 1981 to 8 August 1983), exposure to adolescence (age = 44–55, birth year = 8 August 1983). Prenatal, post-natal and adolescence-exposed groups were considered as early life-exposed groups. In both prenatal and non-exposed stages, participants who were born between September 8, 1986, and August 30, 1987 may have been exposed to famine. Accordingly, these participants were omitted to reduce the misclassification of famine exposure (supplementary file).

The sample size was calculated by applying two population mean formulas using G-Power 3.0.10 and the following assumptions: type one error 5%, 80% power, design effect 1.5, and effect size 0.2. The total estimated sample size was 1396 (1047 exposed early life and 349 non-exposed groups). We included 1384 (1038 early life exposed and 346 unexposed) participants who completed all study variables . The early life exposed group was further divided into prenatal exposed (*n =* 346), post-natal exposed (*n =* 346) and adolescence exposed groups (*n =* 346).

**Fig. 1:**
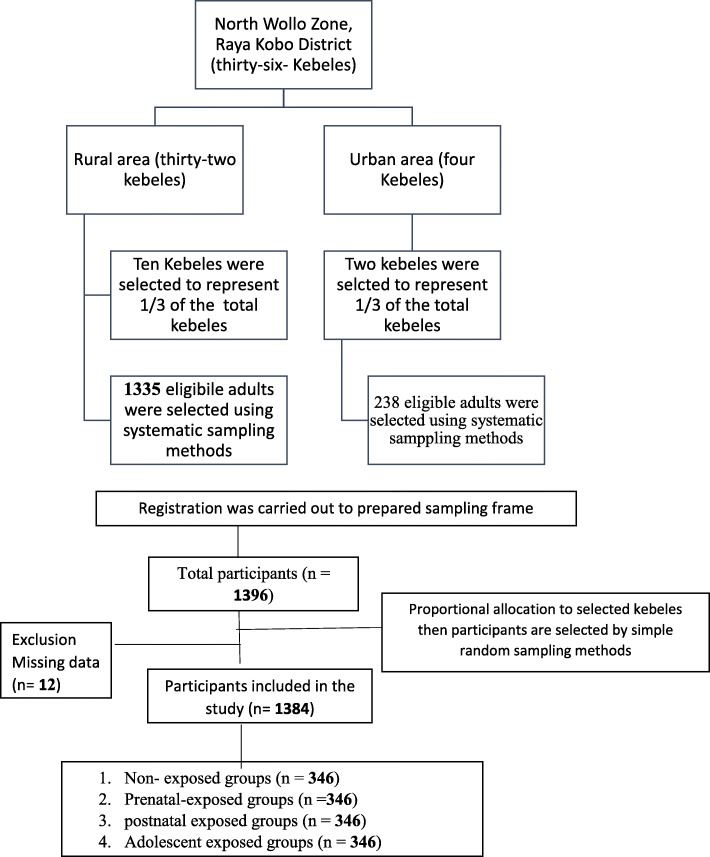
Flow diagram representing sample recruitment

Participants were recruited using a multi-stage stratified sampling technique. Registration was made for all qualified adults. A simple random sampling technique has been used to pick the final participants (Fig. 1). Data was obtained using a pre-tested standardized questionnaire via face-to-face interview. We selected eight qualified clinical nurses to collect socio-demographic, economic and anthropometric data. Adults displaced to other parts of the country during drought and those with physical disabilities, including deformity (Kyphosis, Scoliosis, limb deformity) and pregnant women were excluded from the study.

### Assessment of exposure

Early-life exposure to the 1983–85 Ethiopian famine was the main exposure variable. Participants who were born during famine and/or conceived during famine were considered prenatal exposure. Participants who were 0–2 years of age and 10–19 years of age during famine were deemed to be vulnerable to postnatal and adolescence, respectively. Those who were born 1 year after years of famine were considered as non-exposed category.

### Assessment of outcomes

The outcome of the study included adult height, waist to height ratio, body mass index, overweight, general obesity, and abdominal obesity.

### Anthropometric measurements

Weight was measured in light clothes with bare feet using a beam scale (Seca®, Germany). Standing height was measured using a stadiometer (Seca®, Germany) with the participants touching the vertical stand, and their shoes are taken off. Waist circumference was measured using a waist circumference tape only after breathing out. The hip circumference was measured at the greater trochanter level, with the participants wearing a light cloth right after the waist circumference measurement in a private setting. The accuracy for height, weight, waist and hip circumference was 0.1 cm, 0.1 kg, 0.1 cm, and 0.1 cm, respectively. Following a standard procedure, anthropometric measurements were taken. All measurements were made in triplicate and the mean value was used for further analysis [35]. To ensure the inter-rater reliability, each anthropometric measurement staff was partially retested.

Body mass index (BMI) was defined as the weight in kg divided by height in meters squared (kg/m^2^). Waist to hip ratio (WHR) was defined as waist circumference divided by hip circumference in centimetres. The waist to height ratio (WHtR) was defined as the waist circumference divided by height in centimetres. According to the Ethiopian standard, anthropometric cut-offs for diagnosing obesity among Ethiopian adults, obesity has defined as BMI > 22.2 kg/m^2^, and overweight was defined as 21.6 kg/m^2^ ≤ BMI < 22.2 kg/m^2^ for males. For females, obesity was defined as BMI > 24.5 kg/m^2^, overweight was defined as 21.9 kg/m^2^ ≤ BMI < 23.0 kg/m^2^. Abdominal obesity was defined as waist circumference ≥ 83.7 cm or WHR ≥ 0.88 or WHtR ≥0.49 in males and ≥ 78 cm or WHR ≥ 0.82 or WHtR ≥0.50 in females [36].

### Assessment of co-variables

Information on demographics, socio-economic, lifestyle, food consumption and medical background has been gathered by trained data collecters. The current socio-economic status of the household wealth index has been measured. Wealth index score was generated using the Principal Components Analysis (PCA). The scores for 25 types of assets and utilities were translated into latent factors and the first factor that explained most of the variation was used to group study households into wealth tertiles [37]. Current drinking and smoking status were described as participants who were drinking alcohol and smoking cigarettes for the past 3 months. Every drinker and smoker were described as one who drinks and smokes once in a lifetime, respectively [38]. The International Physical Activity Questionnaire (IPAQ) was used to assess the level of physical activity. After converting to a metabolic equivalent (MET) hour per week and measuring the total MET, the amount of physical activity was transformed to mild, moderate, and intense physical activity [39].

The qualitative food frequency questionnaire (FFQ) consisting of 38 food items covering the key foods consumed in the study area was used to determine dietary habits [40]. In addition, an interview with key informants who know the culture and types of food consumed in the study area was conducted to establish a list of food products. Frequency of consumption of each food per day, per week or per month in the last 1 year as recorded by participants [41]. Adults were then coded as a “consumer” of a food item if they had eaten the food item at least once a week. The dietary pattern was derived using the K-means cluster analysis. Two major dietary patterns were described as healthy and unhealthy dietary pattern based on fruit and vegetable intake. A healthy dietary pattern implies a high consumption of fruit and vegetables, and an unhealthy dietary pattern indicates low consumption of fruit and vegetables.

### Statistical analysis

Epidata version 3.1 was used for entered data. Statistical Package for Social Sciences (SPSS) version 25 ([SPSS Inc., Chicago, Illinois] was used to perform all the statistical analyses. Data were presented as means (±SD) and percentages for continuous and categorical variables, respectively. Continuous variables with skewed distribution were reported as medians (IQR). Group differences were tested using the T-test, ANOVA, and Pearson chi-square test. Bonferroni test was used to conduct multiple comparisons.

Linear regression models were used to estimate β-coefficients and 95% confidence intervals (CI) in order to test associations between early-life famine exposure and adult height, BMI, and waist to height ratio. Multinomial logistic regression analysis was used to investigate the independent impact of early-life exposure on overweight and general obesity in adults. Binary logistic regression analysis were developed to investigate the effects of early-life exposure to famine on abdominal obesity. In both regressions, we first compared participants who were exposed to famine early life with those who were non-exposed to famine. Then, we compared prenatal, post-natal, and adolescence exposed gropus to the non-exposed groups. Crude and adjusted regression models were used to investigate the association between the exposure status of famine (early life, prenatal or post-natal exposure or adolescence) and outcome variables. Crude model includes the outcome variable and exposure status of famine. The adjusted model involves sex, age, residence, educational status, dietary pattern, blood pressure, physical activity level, cigarette smoking, alcohol drinking, history of hypertension and diabetes, and effect modifiers. In addition, a stratified analysis was carried out by sex and residence. A two-sided *P* value < 0.05 was found to be statistically important.

## Results

Table 1 shows background characteristics of the study participants. A total of 1384 (1038 early life exposed and 346 non-exposed) adults had completed data for all variables, and included in the analysis. The mean (±SD) age of early life, prenatal, post-natal, adolescent and non-exposed groups were 40.5 (6.2), 35.1 (0.8), 37.6 (0.5), 48.6 (3.5) and 31.2 (± 0.5) years, respectively. Five-hundred-ninety-one (56.9%) of the participants were females who were exposed to famine in early life. Seven-hundred-seven (68.2%) of the participants were living in rural area and exposed to famine in early life. Four-hundred-eighty-eight (47.2%) of the participants were illiterate and exposed to famine in early life.

**Table 1:** Background characteristics of the study participants according to Ethiopian great famine exposure status, NorthWollo Zone, Raya Kobo district, Northeast Ethiopia, 2019 (*n =* 1384)

Variables	Famine exposure status					*P-* value
	Early life exposed^**a**^ *n =* 1038	Prenatal exposed *n* = 346	Postnatal exposed *n =* 346	Adolescent exposed *n =* 346	Non-exposed *n =* 346	
Age in years, mean ± SD	40.5 ± 6.2	35.1 ± 0.8	37.6 ± 0.5	48.6 ± 3.5	31.20 ± 0.5	< 0.001*
Sex, n (%)						
Female	591 (56.9)	203 (58.6)	207 (59.8)	181 (52.3)	171 (49.4)	< 0.001*
Male	447 (43.1)	143 (41.4)	139 (40.2)	165 (47.7)	175 (50.6)	
Place of residence, n (%)						
Urban	331 (31.8)	110 (31.8)	107 (30.9)	114 (32.9)	102 (29.5)	0.76
Rural	707 (68.2)	236 (68.2)	239 (69.1)	232 (67.1)	244 (70.5)	
Educational status, n (%)						
Illiterates	488 (47.2)	130 (37.5)	163 (47.1)	195 (56.5)	88 (25.5)	< 0.001*
Primary school	256 (24.6)	87 (25.1)	82 (23.6)	87 (25.1)	68 (19.7)	
Secondary school	166 (15.9)	77 (22.3)	56 (16.2)	33 (9.5)	114 (32.9)	
Above secondary	128 (12.3)	52 (15.1)	45 (13.1)	31 (8.9)	76 (21.9)	
Marital status, n (%)						
Single	126 (12.1)	53 (15.3)	53 (15.3)	20 (5.8)	101 (29.2)	< 0.001*
Married	712 (68.6)	228 (65.9)	237 (68.5)	247 (71.4)	223 (64.4)	
Divorced/widowed	200 (19.3)	65 (18.8)	56 (16.2)	79 (22.8)	22 (6.4)	
Physical activity level, n (%)						
Low	149 (14.4)	31 (8.9)	53 (15.3)	65 (18.8)	29 (8.4)	0.145
Moderate	255 (24.5)	76 (21.9)	97 (28.1)	82 (23.6)	75 (21.7)	< 0.001*
Vigorous	634 (61.1)	239 (69.2)	196 (56.6)	199 (57.6)	242 (69.9)	
Dietary pattern, n (%)						
Healthy	486 (46.8)	171 (49.4)	155 (44.8)	160 (46.3)	167 (48.3)	0.36
Unhealthy	552 (53.2)	175 (50.6)	191 (55.2)	186 (53.7)	179 (51.7)	
Wealth index, n (%)						
Low SES	291 (28.2)	127 (36.7)	121 (34.9)	43 (12.4)	145 (41.9)	< 0.001*
Medium SES	333 (32.0)	158 (45.6)	129 (37.3)	46 (13.3)	152 (43.9)	
High SES	414 (39.8)	61 (17.7)	96 (27.8)	257 (74.3)	49 (14.2)	
Current smoker,n (%)						
Yes	20 (1.9)	9 (2.6)	6 (1.7)	5 (1.4)	10 (2.8)	0.05*
No	1018 (98.1)	337 (97.4)	340 (98.3)	341 (98.5)	336 (97.2)	
Current drinker, n (%)						
Yes	522 (50.3)	224 (64.7)	165 (47.7)	133 (38.5)	236 (68.2)	< 0.001*
No	516 (49.7)	122 (35.3)	181 (52.3)	213 (61.5)	110 (31.8)	

^a^Prenatal, post-natal and adolescence exposed, *P-*value—represents Independent samples t-tests for continuous variables or χ2-test for categorical variables, SES – socio-economic status,* Statistical significance

### Anthropometric outcomes

**Fig. 2:**
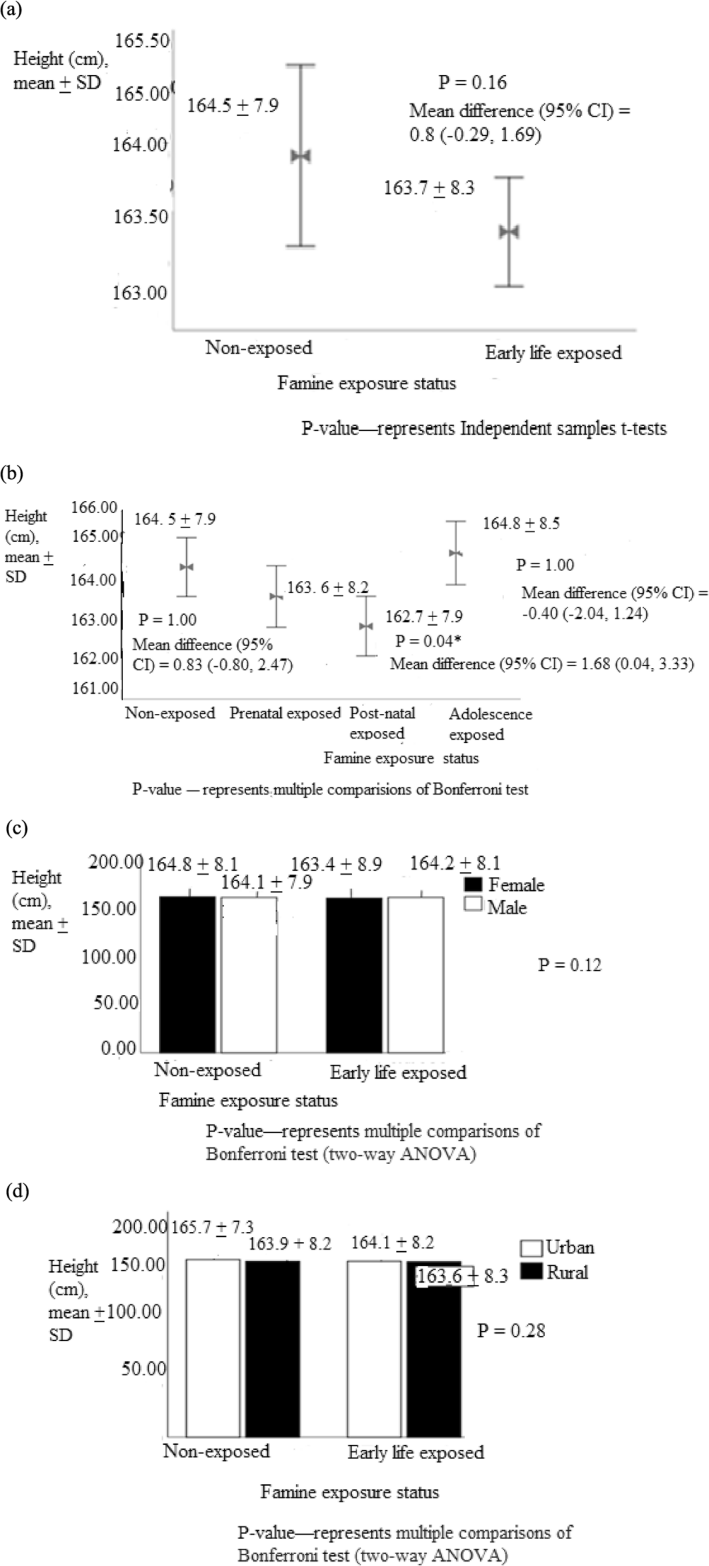
Adult height among (**a**) early life exposed (**b**) prenatal, post-natal and adolescence exposed groups (**c**) grouped by sex and (**d**) residency, North Wollo Zone, Raya Kobo district, Northeast Ethiopia, 2019 (*n* = 1384)

The mean (± SD) height among early life, prenatal, post-natal, adolescence exposed and non-exposed groups were 163.7 ± 8.3, 163.6 ± 8.2, 162.7 ± 7.9, 164.8 ± 8.5 and 164.5 ± 7.9 cm, respectively. The mean height of females and males exposed groups were 163.4 ± 8.4 and 164.2 ± 8.0, respectively, while it was 164.1 ± 8.2 for urban and 163.6 ± 8.3 for rural residents (Fig. 2).

**Fig. 3:**
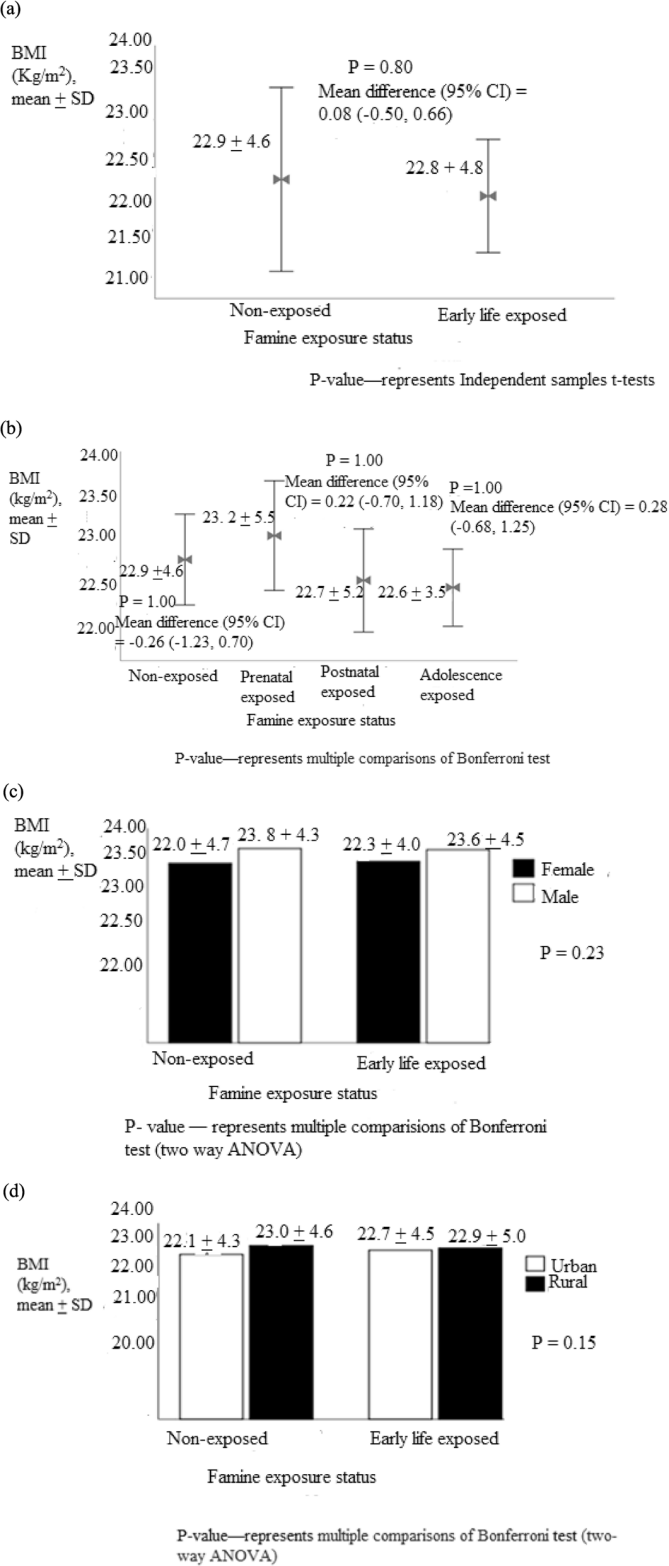
Body mass index among (**a**) early life exposed (**b**) prenatal, post-natal and adolescence exposed groups (**c**) grouped by sex and (**d**) residency, North Wollo Zone, Raya Kobo district, Northeast Ethiopia, 2019 (*n =* 1384)

**Fig. 4:**
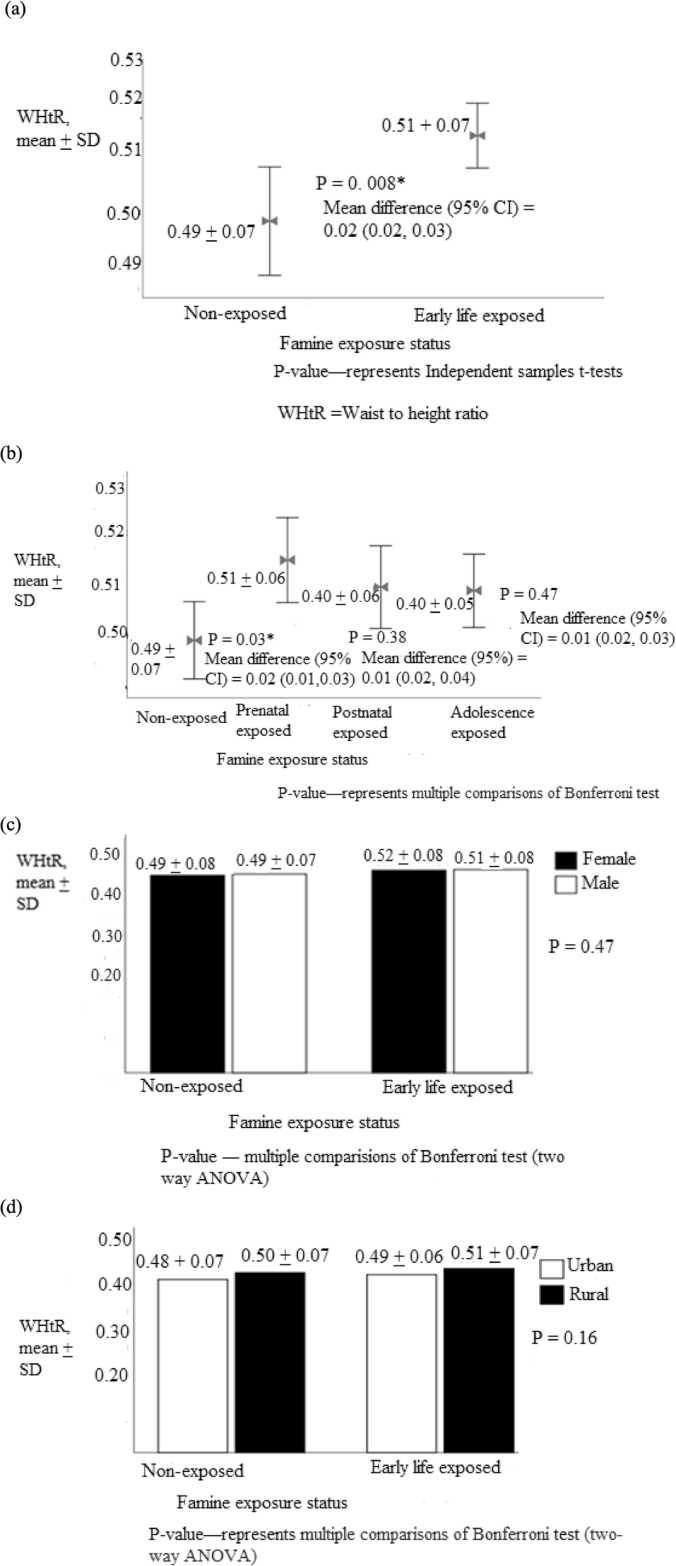
Waist to height ratio among (**a**) early life exposed (**b**) prenatal, post-natal and adolescence exposed groups (**c**) grouped by sex and (**d**) residency, North Wollo Zone, Raya Kobo district, Northeast Ethiopia, 2019 (*n =* 1384)

Similarly, the mean BMI was 22.8 ± 4.8, 23.2 ± 5.5, 22.7 ± 5.2, 22.6 ± 3.5 and 22.9 ± 4.6 kg/m^2^ in early life, prenatal, post-natal, adolescent exposed and non-exposed groups, respectively **(**Fig. 3). Similarly, the mean of BMI in early life, prenatal, post-natal, adolescent exposed and non-exposed groups were 22.8 ± 4.8, 23.2 ± 5.5, 22.7 ± 5.2, 22.6 ± 3.5, and 22.9 ± 4.6 kg/m^2^_,_ respectively **(**Fig. 3**)**. The mean waist to height ratio in early life, prenatal, post-natal, adolescent exposed and non-exposed were 0.51 ± 0.07, 0.51 ± 0.06, 0.40 ± 0.06, 0.40 ± 0.05 and 0.49 ± 0.07, respectively (Fig. 4).

The magnitude of overweight in early life, prenatal, post-natal, adolescent exposed, and non-exposed were 15.4, 14.2, 13.2, and 18.8% and 14.6%, respectively. Likewise, the prevalence rate of general obesity in early life, prenatal, post-natal, adolescent exposed, and non-exposed was 8.8, 22.4, 1.2, and 1.9% and 22.0%, respectively. The prevalence of abdominal obesity was 32.5, 29.7, 31.9, 35.8, and 30.9% in early life exposed, prenatal exposed, post-natal exposed, adolescent exposed, and non-exposed groups, respectively **(**Table 2**)**.

**Table 2:** Magnitude of adulthood obesity, overweight and abdominal obesity by famine exposure status, sex of participant and residency, Wollo Zone, Raya Kobo district, Northeast Ethiopia, 2019 (*n =* 1384)

Famine exposure status						
Variables	Early life exposed^a^	Prenatal exposed	Postnatal exposed	Adolescence exposed	Non-exposed	*P-*value
Overweight, n (%)	159 (15.4)	49 (14.2)	45 (13.2)	65 (18.8)	51 (14.6)	0.05
Sex, n (%)						
Female	80 (13.5)	20 (9.9)	27 (13.0)	33 (18.2)	16 (9. 4)	0.70
Male	79 (17.9)	29 (28.6)	18 (13.3)	32 (19.4)	35 (9.4)	
Residence (%)						
Urban	58 (17.9)	15 (14.0)	17 (16.5)	26 (22.8)	10 (9.8)	0.67
Rural	101 (14.3)	34 (14.3)	28 (11.7)	39 (16.8)	41 (16.5)	
General Obesity, n (%)	91 (8.8)	77 (22.4)	(1.2)	10 (1.9)	77 (22.0)	< 0.001*
Sex, n (%)						
Female	53 (9.0%)	47 (23.0)	2 (1.0)	4 (2.0)	46 (27.1)	< 0.001*
Male	38 (8.6%)	30 (21.3)	2 (1.5)	6 (3.6%)	31 (17.2)	
Residence, n (%)						
Urban	32 (9.9)	23 (22.5)	1 (1.0)	9 (7.9)	23 (22.5)	< 0.001*
Rural	59 (8.3)	55 (23.7)	3 (1.3)	1 (0.1)	54 (21.8)	
Abdominal obesity, n (%)	335 (32.5)	102 (29.7)	109 (31.9)	124 (35.8)	108 (30.9)	0.57
Sex, n (%)						
Female	179 (30.3)	59 (29.1)	64 (30.9)	56 (30.9)	53 (31.2)	0.82
Male	156 (35.4)	43 (30.5)	45 (33.3)	68 (41.2)	55 (30.6)	
Residence, n (%)						
Urban	119 (36.7)	32 (29.9)	35 (34.0)	52 (45.6)	33 (32.4)	0.42
Rural	216 (30.5)	70 (29)	74 (31.0)	72 (31.0)	75 (30.2)	

^a^Prenatal, post-natal and adolescence exposed, *P-*value—represents χ2-test for categorical variables, * Statistical significance

### **Association of** famine exposure in early life and anthropometric outcomes in adults

The association between famine exposure to Ethiopian great famine and anthropometric measurements in adults is described in Table 3. Linear regression analysis was used to analyse the association between anthropometric measurements and the famine exposure in early life. Adjusted for covariates, early life exposure to famine showed 1.83 (β = − 1.83; 95% CI: − 3.05, − 0.58) cm decreased height of adults compared to non-exposed group. Based on time of exposure to famine, height of adults decreased by 1.35 (β = − 1.35; 95% CI: − 2.56, − 0.14) cm and 2.07 (β = − 2.07; 95% CI: − 3.31, − 0.80) cm after prenatal and post-natal exposure to famine, respectively. However, exposure to famine during adolescence was not associated with adult height (β = − 1.66; 95% CI: − 5.95, 2.63). Likewise, famine exposure during early life was not associated with BMI in adults (β = 0.45; 95% CI: − 0.13, 1.04) (adjusted model). Based on time of exposure to famine, prenatal (β = 0.37; 95% CI: − 2.35, 1.62), post-natal (β = 0.38; 95% CI: − 0.35, 1.13) and adolescence (β = 0.23; 95% CI: − 0.44, 0.93) was not associated with BMI in adults. In this study, early life exposure to famine was associated with increased waist to height ratio in adults by 0.02 (β = 0.02; 95% CI: 0.01, 0.03) points in the full adjusted model. Based on time of exposure to famine, prenatal and post-natal exposure to famine resulted in 0.03 (β = 0.03; 95% CI: 0.02, 0.03) and 0.01 (β = 0.01; 95% CI: 0.01, 0.02) increased in waist to height ratio, respectively. Famine exposure during adolescence was not associated with increased waist to height ratio (β = 0.01; 95% CI: - 0.02, 0.02).

**Table 3:** Associations offamine exposure and anthropometric measurements, in adults, North Wollo Zone, Raya Kobo district, Northeast Ethiopia, 2019 (*n =* 1384)

Measurements	Famine exposure status				
Height^c^	Early life exposed	Prenatal exposed	postnatal exposed	Adolescence Exposed	Non-exposed
Crude model^a^	−0.70 (−1.69, 0.29)	−2.04 (−2.74, 0.37)	−1.67 (−2.86, −0.50)	0.40 (−0.83, −1.65)	Ref.
Adjusted model^b^	−1.83 (−3. 05, 0.58)	−1.35 (−2.56, −0.14)	−2.07 (−3.31, −0.80)	−1.66 (−5.95, 2.63)	Ref.
Waist to height ratio (WHtR)					
Crude model	0.01 (0.01,0.02)	0.02 (0.01, 0.03)	0.01 (− 0.01, 0.02)	0.01 (− 0.01, 0.02)	Ref.
Adjusted model	0.02 (0.01,0.03)	0.03 (0.02, 0.03)	0.01 (0.01, 0.02)	0.01(−0.02, 0.02)	Ref.
Body mass index					
Crude model	0.08 (−0.67, 0.49)	0.25 (−0.50, 1.01)	0.22 (− 0.94, 0.50)	0.29 (− 0.93, 0.33)	Ref.
Adjusted model	0.45 (−0.14, 1.04)	0.37 (−2.35, 1.62)	0.38 (− 0.35, 1.13)	0.23 (− 0.44, 0.93)	Ref.

*Ref* Reference

Data are β-coefficients (95% confidence interval) from multiple linear regression analysis

All β-coefficients are related to the non-exposed groups

^a^Measurements with famine exposure status (unadjusted for any covariate)

^b^Adjusted for sex, age, residence, educational status, wealth index, dietary pattern, increased blood pressure, physical activity, cigarette smoking, alcohol drinking, history of chronic diseases and effect modifiers

^c^*Adult height adjusted for sex, age, residence, educational status, wealth index and effect modifiers (*Adjusted model*)*

On the adjusted model of multinomial logistic regression analysis, the odds of overweight in males among early life, prenatal, post-natal and adolescence exposed group were (OR: 0.82, 95% CI (0.54,1.23)), (OR: 1.10, 95% CI (0.65,1.85)), (OR: 0.89, 95% CI (0.58, 5.36)), (OR: 0.36, 95% CI (0.07, 1.65)), respectively. The odds of general obesity in males were (OR:1.01, 95% CI (0.60,1.68)), (OR:0.80, 95% CI (0.23, 3.58)), (OR:0.59, 95% CI (0.03, 10.39)), (OR:0.45, 95% CI (0.09,2.13)), among early life, prenatal, post-natal and adolescence exposed groups in males, respectively (Table 4). In females, the odds of having overweight were (OR:0.84, 95% CI (0.56,1.26)), (OR:1.16, 95% CI (0.39, 3.40)), (OR:2.79, 95% CI (0.28, 27.5)), (OR:1.64, 95% CI (0.39, 6.85)) among early life, prenatal, postnatal and adolescence exposed, respectively. The odds of general obesity were (OR:0.85, 95% CI (0.55,1.33)), (OR:1.04, 95% CI (0.33, 3.30)), (OR:0.20, 95% CI (0.01, 2.30)), (OR:2.32, 95% CI (0.45,13.0)), respectively (Table 5).

**Table 4:** Association of famine exposure in early life with overweight and general obesity among males, multinomial logistic regression analysis, North Wollo Zone, Raya Kobo district, Northeast Ethiopia, 2019 (*n* = 1384)

Famine exposure status						
Models	*BMI category*	Variables	Early life exposed ^**ð**^	Prenatal exposed	Post-natal exposed	Adolescence exposed
			OR (95% CI)	OR (95% CI)	OR (95% CI)	OR (95% CI)
Crude model	Overweight	Exposed	1.09 (0.80,1.51)	0.97 (0.66, 1.41)	0.34 (0.04, 3.01)	2.78 (0.59,12.9)
	Obesity	Exposed	0.82 (0.54,1.23)	1.10 (0.65,1.85)	0.89 (0.58, 5.36)	0.36 (0.07, 1.65)
Adjusted model	Overweight	Exposed	1.05 (0.70,1.57)	0.44 (0.53, 1.23)	0.41 (0.05, 3.73)	2.75 (0.58,12.8)
		Female	0.98 (0.73,1.34)	1.07 (0.70, 1.63)	1.29 (0.05,1.09)	0.72 (0.46,1.2)
		Age	0.97 (0.95,1.01)	0.78 (0.61,1.02)	0.84 (0.59,1.17)	1.04 (0.95,1.13)
		Urban	1.09 (0.80,1.50)	1.24 (0.81,1.92)	1.05 (0.67, 1.64)	1.26 (0.81, 1.97)
		Illiterate	2.03 (1.24,3.29)^a^	2.32 (1.26,4.25) ^a^	2.42 (1.24, 4.67) ^a^	1.41 (0.72,2.76)
		Primary school	1.39 (0.84,2.34)	0.87 (0.44, 1.74)	1.80 (0.90, 3.59)	1.05 (0.52,2.15)
		Secondary school	1.26 (0.75,2.12)	1.23 (0.67,2.26)	1.55 (0.80, 3.00)	1.20 (0.62,2.34)
		Unhealthy dietary pattern	1.05 (0.78,1.38)	0.97 (0.66, 1.44)	0.94 (0.63, 1.40)	1.03 (0.68,1.55)
		Low physical activity level	1.06 (0.68,1.63)	0.63 (0.28, 1.36)	1.44 (0.79, 2.74)	0.86 (0.46, 1.62)
		Moderate activity level	0.95 (0.67,1.34)	1.11 (0.69, 1.77)	1.39 (0.87, 2.22)	0.96 (0.58,1.59)
		Current drinker	0.96 (0.71,1.30)	0.43 (0.17,1.07)	0.64 (0.36, 1.13)	0.29 (0.06,1.42)
		Current smoker	0.92 (0.53,1.56)	0.64 (0.36,1.13)	0.43 (0.17, 1.07)	0.53 (0.29,1.00)
	Obesity	Exposed	1.01 (0.60,1.68)	0.80 (0.23, 3.58)	0.59 (0.03, 10.39)	0.45 (0.09,2.13)
		Female	0.63 (0.43,0.91) ^a^	0.44 (0.25, 0.78) ^a^	0.55 (0.32, 0.94) ^a^	0.67 (0.40,1.11)
		Age	1.02 (0.99,1.06)	0.94 (0.66,1.33)	0.93 (0.61, 1.45)	0.96 (0.80,1.05)
		Urban	0.73 (0.48,1.09)	0.86 (0.46, 1.59)	0.62 (0.34, 1.14)	0.74 (0.43,1.25)
		Illiterate	1.56 (0.88,2.77)	1.91 (0.86,4.25)	2.46 (1.05, 5.41) ^a^	2.43 (1.06,5.55) ^a^
		Primary school	1.15 (0.63,2.12)	0.96 (0.40,2.33)	1.57 (0.67, 3.70)	1.73 (0.73, 4.09)
		Secondary school	1.43 (0.79,2.54)	1.18 (0.56,2.49)	1.87 (0.87, 4.05)	1.42 (0.59,3.38)
		Unhealthy dietary pattern	0.97 (0.69,1.37)	1.09 (0.65, 1.85)	0.98 (0.60, 1.63)	1.20 (0.75,1.92)
		Low physical activity level	1.42 (0.86,2.32)	1.12 (0.48, 2.59)	1.71 (0.82, 3.60)	1.05 (0.53, 2.08)
		Moderate activity level	1.35 (0.90,2.03)	0.87 (0.44,1.75)	1.78 (1.00, 3.12)	1.33 (0.77, 2.29)
		Current drinker	1.50 (0.82, 1.61)	1.17 (0.58, 2.36)	0.23 (0.02, 2.47)	0.48 (0.23,1.00)
		Current smoker	1.20 (0.88, 1.61)	1.50 (0.73,3.10)	0.78 (0.46, 1.35)	0.29 (0.06, 1.42)

Normal BMI, Male gender, rural residence, healthy dietary pattern, vigorous, non-smoker/drinker, physical activity above education secondary school education, healthy dietary pattern was reference

^a^Variables with significant association

**Table 5:** Association of famine exposure in early life with overweight and general obesity among females, multinomial logistic regression analysis, North Wollo Zone, Raya Kobo district, Northeast Ethiopia, 2019 (*n* = 1384)

Famine exposure status						
Models	*BMI category*	Variables	Early life exposed ^**ð**^	Prenatal exposed	Post-natal exposed	Adolescence exposed
			OR (95% CI)	OR (95% CI)	OR (95% CI)	OR (95% CI)
Crude model	Overweight	Exposed	0.96 (0.70, 1.32)	0.96 (0.65, 1.42)	0.95 (0.65, 1.41)	0.98 (0.66, 1.44)
	Obesity	Exposed	1.08 (0.76, 1.53)	0.91 (0.60,1.40)	1.13 (0.74, 1.75)	1.24 (0.80, 1.90)
Adjusted model	Overweight	Exposed	0.84 (0.56,1.26)	1.16 (0.39, 3.40)	2.79 (0.28, 27.5)	1.64 (0.39, 6.85)
		Female	0.79 (0.58,1.06)	0.65 (0.43, 1.00)	0.72 (0.47,1.10)	0.75 (0.48,1.15)
		Age	0.99 (0.96,1.02)	1.08 (0.83,1.40)	1.19 (0.84,1.69)	1.02 (0.94,1.10)
		Urban	0.85 (0.62,1.17)	0.83 (0.51,1.33)	0.80 (0.49, 1.28)	0.70 (0.44, 1.00)
		Illiterate	0.75 (0.47,1.16)^a^	0.58 (0.32,1.06) ^a^	0.80 (0.42 1.52)	0.80 (0.42, 1.54)
		Primary school	0.86 (0.54,1.38)	0.74 (0.39, 1.40)	0.80 (0.42, 1.55)	0.78 (0.39, 1.55)
		Secondary school	1.05 (0.66,1.66)	0.73 (0.42,1.29)	1.17 (0.64, 2.20)	1.01 (0.53, 1.92)
		Unhealthy dietary pattern	0.97 (0.74,1.28)	1.13 (0.76, 1.69)	0.81 (0.54, 1.20)	1.88 (0.59,1.30)
		Low physical activity level	0.69 (0.45,1.07)	0.76 (0.36, 1.60)	0.48 (0.24, 0.98)	0.59 (0.32, 1.11)
		Moderate activity level	0.48 (0.60,1.17)	0.68 (0.41, 1.13)	1.18 (0.74, 1.90)	0.65 (0.39,1.06)
		Current drinker	0.85 (0.54,1.36)	0.54 (0.34,1.25)	0.75 (0.50, 1.12)	0.65 (0.31,1.20)
		Current smoker	0.98 (0.61,1.57)	0.65 (0.37,1.12)	0.90 (0.43, 2.22)	0.30 (0.31, 1.20)
	Obesity	Exposed	0.85 (0.55,1.33)	1.04 (0.33, 3.30)	0.20 (0.01, 2.30)	2.32 (0.45,13.0)
		Female	0.68 (0.58,0.54)^a^	0.57 (0.37, 0.92) ^a^	0.51 (0.32, 0.82) ^a^	0.60 (0.37,0.97)
		Age	0.99 (0.96,1.02)	1.05 (0.80,1.39)	1.17 (1.15, 2.52) ^a^	1.04 (0.95,1.13)
		Urban	1.15 (0.82,1.62)	1.59 (1.00, 2.53) ^a^	1.09 (0.66, 1.180)	0.90 (0.55,1.48)
		Illiterate	0.61 (0.37, 1.00)	0.56 (0.30,1.10)	0.97 (0.48, 1.96)	0.54 (0.27, 1.09)
		Primary school	0.99 (0.61,1.63)	0.88 (0.47, 1.68)	0.90 (0.45, 1.83)	0.81 (0.41, 1.61)
		Secondary school	0.78 (0.47,1.29)	0.51 (0.26,0.96) ^a^	0.77 (0.39, 1.55)	0.58 (0.29, 1.18)
		Unhealthy dietary pattern	0.82 (0.60,1.12)	0.79 (0.52, 1.21)	0.65 (0.42, 1.00)	0.80 (0.52,1.25)
		Low physical activity level	0.09 (0.42, 1.13)	1.06 (0.51, 2.20)	0.59 (0.28, 1.27)	0.76 (0.39, 1.49)
		Moderate activity level	0.97 (0.67,1.39)	1.04 (0.63,1.71)	1.34 (0.80, 2.25)	0.83 (0.49, 1.41)
		Current drinker	0.84 (0.57, 1.24)	0.16 (0.01, 2.25)	0.24 (0.01, 5.30)	0.70 (0.36,1.70)
		Current smoker	1.86 (0.56, 1.32)	0.64 (0.35,1.17)	0.28 (0.56, 3.41)	0.81 (0.53, 1.30)

Normal BMI, Male gender, rural residence, healthy dietary pattern, vigorous, non-smoker/drinker, physical activity above education secondary school education, healthy dietary pattern was reference

^a^Variables with significant association

On the multivariable logistic regression, the odds of abdominal obesity among early life, prenatal, post-natal and adolescence exposed group were (OR:0.90, 95% CI (0.76, 1.22)), (OR:0.88, 95% CI (0.62, 1.27)), (OR:0.99, 95% CI (1.36, 7.30)), (OR:1.14, 95% CI (0.32, 4.02)) in males and females (OR:0.95, 95% CI (0.65, 1.39)), (OR:0.51, 95% CI (0.20, 1.40)), (OR:1. 04, 95% CI (0.69, 1.53)), (OR:0.87, 95% CI (0.55, 1.38)), respectively (Table 6).

**Table 6:** Associations of early life famine exposure and abdominal obesity among adults stratified by sex, North Wollo Zone, Raya Kobo district, Northeast Ethiopia, 2019 (*n =* 1384)

Famine exposure status					
Models	Abdominal obesity	Early life exposed ^**ð**^	Prenatal exposed	Post-natal exposed	Adolescence exposed
		OR (95% CI)	OR (95% CI)	OR (95% CI)	OR (95% CI)
Crude model	Male	0.97 (0.73,1.28)	0.93 (0.65, 1.32)	0.91 (0.64, 1.28)	1.10 (0.76, 1.52)
	Female	1.12 (0.82, 1.50)	1.00 (0.69,1.46)	1.03 (0.71, 1.50)	1.30 (0.91, 1.86)
Adjusted model	Male	0.90 (0.76, 1.22)	0.88 (0.62, 1.27)	0.99 (1.36, 7.30)	1.14 (0.32, 4.02)
	Female	0.95 (0.65, 1.39)	0.51 (0.20, 1.40)	1.04 (0.69, 1.53)	0.87 (0.55, 1.38)

*All odd ratios are related to the non-exposed groups*

*Ref* Reference, *Cl* Confidence interval, *OR* Odds ratio, * Statistical significance

*Crude model: Famine exposure in different stage of life and abdominal obesity*

*Adjusted model:* adjusted for age, educational status, wealth index, dietary pattern, increased blood pressure, physical activity, cigarette smoking, alcohol drinking, history of chronic diseases and effect modifiers

Table 7 shows the effect of famine exposure in early life on adulthood anthropometry, which was not modified by sex (famine exposure * sex) and residence (famine exposure *residence) (*p interaction > 0.05).*

**Table 7:** Associations of early life famine exposure and adulthood anthropometric measurements stratified by sex and residency, multivariable linear regression analysis, North Wollo Zone, Raya Kobo district, Northeast Ethiopia, 2019 (*n =* 1384)

Height^a^	Famine exposure status				
	Early life exposed	Prenatal exposed	post-natal exposed	Adolescence Exposed	Non-exposed
Male	−4.67 (−6.36, −2.97)	−1.44 (−2.86, −0.03)	−1.42 (−3.05, 0.21)	−1.1 (− 1.30, −4.83)	Ref.
Female	− 0.16 (− 4.56, 4.24)	− 2.73 (− 4.45, − 1.02)	− 2.30 (− 4.05, − 0.53)	− 1.60 (− 3.05, 4.16)	Ref.
Urban	−0.36 (− 1.56, 0.82)	0.07 (− 3.82, − 3.97)	0.98 (− 2.42, 0.45)	0.45 (− 6.04, 5.13)	Ref.
Rural	−4.24 (− 6.39, − 2.09)	−2.47 (− 4.63,0.31)	− 3.33 (−5.43, − 1.24)	−2.28 (−9.15, 4.72)	Ref.
Waist to height ratio (WHtR)					
Male	0.02 (0.02,0.04)	0.02 (0.01, 0.03)	0.02 (0.01, 0.04)	0.07 (0.03, 0.04)	Ref.
Female	0.07(− 0.08,0.02)	0.02 (0.04 0.05)	0.02 (0.02, 0.05)	0.02 (0.03, 0.04)	Ref.
Urban	0.03 (0.02, 0.05)	0.03 (0.03,0.054)	0.10 (−0.20, 0.02)	0.03 (− 0.09, 0.03)	Ref.
Rural	0.07 (−0.06, 0.02)	0.03 (0.02, 0.03)	0.07(−0.12, 0.05)	0.05 (−0.05, 0.05)	Ref.
Body mass index					
Male	−0.15 (− 0.93, 0.63)	−0.20 (− 1.20, 0.80)	0.20 (−2.4, 2.7)	− 1.36 (− 2.27, 0.52)	Ref.
Female	0.23 (− 0.62, 1.07)	0.84 (− 0.19, 1.88)	0.16 (− 0.89, 1.22)	0.16(− 0.89, 1.22)	Ref.
Urban	−0.34(− 1.05, 0.37)	−0.07(− 0.99, 0.84)	−0.58(− 1.42, 0.38)	−0.37(− 1.15, 0.41)	Ref.
Rural	0.56(−0.44, 1.56)	1.06 (− 0.25, 2.38)	0.65 (− 0.60, 1.92)	0.02 (− 1.01, 1.04)	Ref.

All β-coefficients are related to the non-exposed groups, *Ref—Reference*

All analyses were adjusted for age, educational status, wealth index, dietary pattern, increased blood pressure, physical activity, cigarette smoking, alcohol drinking, history of chronic diseases and effect modifiers

^a^*Adult height adjusted for sex, age, residence, educational status, wealth index and effect modifiers*

## Discussion

In this study, height and waist to height ratio showed an inverse and linear association with early-life exposure to famine, respectively. On average, a reduction by 1.83 cm of adult height was seen among early life exposed groups compared to non-exposed groups. Likewise, early life exposure to famine was associated with increased waist to height ratio in adults by 0.02. However, we could not find an association between early-life exposure to famine and BMI in adults. In linear regression analysis, we identified the timing of exposure to famine (prenatal, post-natal, or adolescence) as an independent predictor. Only prenatal and post-natal exposure to famine resulted in a 1.35 and 2.07 cm decrease in adult height, respectively. Similarly, prenatal and post-natal exposure to famine were associated with 0.03 and 0.01 increase in waist to height ratio, respectively. Exposure to famine in early life was not associated with increased risk of overweight, general obesity, and abdominal obesity.

The finding that early-life exposure to famine associated with a shorter adult height was consistent with previous famine studies [29, 33, 42, 43]. This finding corroborates Barker et al., theoretical explanations that consider early life undernutrition as the most important factor yielding long-term consequences on an individual’s growth and development [19]. Moreover, the accumulation of risk model also suggests that health conditions in later life is resulted not only from a single period of exposure to famine, but also from exposures during prenatal, infancy, and adolescence [44].

In our study, we did not found any effect on adult height from exposure to famine during adolescence. This can be seen from the psychosocial enhancement dimensions of childhood and social growth change, as stature is a summary of all previous height increments [45]. Adolescence can alter the height of a peer group due to psychosocial stimulation. Eveidences showed that adequate early-life psychosocial enhancement is well known to be associated with growth control [45–47]. As a result, the effect of undernutrition on early life can be reversed to an adjusted body height [45, 46].

An increased waist to height ratio among adults was observed in the present study after early-life exposure to famine. The finding was consistent with other studies conducted in China [31], Dutch [26], and Nigeria [48]. This could be due to intrauterine nutritional insult, which brings developmental adaptations that permanently change the body structure and function and increases the risk of intra-abdominal fat deposition in later life [22]. Nonetheless, our study was not supported by studies conducted in Leningrad [49, 50]. Furthermore, our study revealed that famine exposure during adolescence was not associated with increased risk of the waist to height ratio. This could indicates that the increased risk of waist to height ratio in later life is programed during the period of intrauterine and first 2 years of post-natal life [51].

In this study, exposure to famine during early life was not associated with BMI in adults. The finding is in line with earlier famine studies [48, 50]. However, it is not supported by famine studies conducted in China [25, 33, 42] and Dutch [28]. This could be due to the difference in dietary habit, age of participant and ethnicity [52, 53]. Moreover, adulthood BMI is mainly related to current dietary intake. Monotonous low-quality diets with inadequate animal products, fresh fruits, and vegetables is the norm in Ethiopia [54]. This could have resulted in chronic energy deficiency (CED), which is explained by decreased BMI [55].

Furthermore, our study attested that exposure to famine during the early stage of life was not significantly associated with overweight, general obesity, and abdominal obesity in adults. This finding was in line with previous studies conducted in Europe [28, 49, 50] and Asia [56, 57]. Nevertheless, it was not consistent with other Chinese famine studies [30, 33]. This could imply that the impact of famine on adulthood overweight/obesity could be exacerbated by lifestyle factors including smoking, unhealthy diets, and sedentary lifestyles [31]. The study of Zhou J et al., (2019) in Chinese study observed that the joint effect between famine and harmful dietary pattern could have serious consequences on later-life health outcomes [58].

We also found a decreased in adult height, increased waist to height ratio and body mass index among female survivors than males similar to previous studies [28, 29, 31, 33]. This could be due to the mortality selection hypothesis, which indicates undernutrition in utero costs for female babies than males resulting in a better opportunity to survive in the harsh environment for female babies [59]. In Ethiopia, the culture of son preference and discrimination against girls often leads to unequal distribution of food, which may augment these risk markers [60].

The finding of this study implies nutrition during early stage of human growth and development has a significant effect on adult health. It also infers proper feeding of mothers and children will allow future generations give a child with a good start to life. The sex difference impact of undernutrition in early life indicates that the severity of malnutrition among females is high, and female babies need a better nutritional care across the life span.

Our study acknowledges the following limitations. First, the findings might be partly biased by self-selection effects. It is plausible that potential participants couldn’t participate because of health-related problems, and others may have already died due to the exposure. Second, other proxy indicators of food restrictions in early life such as birth weight and childhood eviromental conditions were not captured. These all might underestimate the true relationship between exposure and the outcome. Third, the long-term consequence of famine is more likely depends on the severity of the exposure within the individual households, which could not be measured due the widspread nature of the famine.

## Conclusions

Prenatal and post-natal exposure to famine was associated with height deficits and increased waist to height ratio in adults. Therefore, these findings highlight the need to improve diet during pregnancy and early childhood life as these periods are critical for the origin of adulthood disease. Further studies with better controls for age, famine severity, and consideration of individual food intakes are needed to estimate the long term health effect of famine.

## Supplementary Information

**Additional file 1: Table 1**: Window of exposure to the 1983–85 Ethiopian Great Famine birth cohorts, Raya Kobo District, Northeast Ethiopia, 2019.

## Data Availability

The data used and/or analyzed during the current study are available fromthe corresponding author on request.

## Abbreviations

BMI: Body mass index
DOHaD: Developmental Origins of Health and Disease
FOAD: Fetal Origin of Adult Diseases
WHtR: Waist to height ratio

## References

[1] Jelliffe DB, Organization WH. The assessment of the nutritional status of the community (with special reference to field surveys in developing regions of the world): World Health Organization; 1966. 4960818

[2] Apovian CM (2016). Obesity: definition, comorbidities, causes, and burden. Am J Manag Care.

[3] Hu FB (2007). Obesity and mortality: watch your waist, not just your weight. Arch Intern Med.

[4] Dobbelsteyn CJ, Joffres MR, MacLean DR, Flowerdew G (2001). A comparative evaluation of waist circumference, waist-to-hip ratio and body mass index as indicators of cardiovascular risk factors. The Canadian Heart Health Surveys. Int J Obes.

[5] Martin L, Collin J (2015). An introduction to growth and atypical growth in childhood and adolescence. Nurs Child Young People.

[6] Dietz WH. Periods of risk in childhood for the development of adult obesity--what do we need to learn? J Nutr. 1997;127(9):1884s–6s. 10.1093/jn/127.9.1884S 9278575

[7] Langley-Evans SC (2015). Nutrition in early life and the programming of adult disease: a review. J Hum Nutr Diet.

[8] Sgarbieri VC, Pacheco MTB. Human development: from conception to maturity. Braz J Food Technol. 2017;20(0).

[9] Horton S, Steckel RH (2013). Malnutrition: global economic losses attributable to malnutrition 1900–2000 and projections to 2050. How Much Have Global Problems Cost the Earth? A Scorecard from 1900 to.

[10] Benonisdottir S, Oddsson A, Helgason A, Kristjansson RP, Sveinbjornsson G, Oskarsdottir A (2016). Epigenetic and genetic components of height regulation. Nat Commun.

[11] Silventoinen K (2003). Determinants of variation in adult body height. J Biosoc Sci.

[12] Hruby A, Manson JE, Qi L, Malik VS, Rimm EB, Sun Q (2016). Determinants and consequences of obesity. Am J Public Health.

[13] Bozzoli C, Deaton A, Quintana-Domeque C (2009). Adult height and childhood disease. Demography..

[14] Yang J, Benyamin B, McEvoy BP, Gordon S, Henders AK, Nyholt DR (2010). Common SNPs explain a large proportion of the heritability for human height. Nat Genet.

[15] Matthews SG (2002). Early programming of the hypothalamo-pituitary-adrenal axis. Trends Endocrinol Metab.

[16] Black RE, Victora CG, Walker SP, Bhutta ZA, Christian P, de Onis M (2013). Maternal and child undernutrition and overweight in low-income and middle-income countries. Lancet (London, England).

[17] Dewey KG, Begum K (2011). Long-term consequences of stunting in early life. Matern Child Nutr.

[18] Alter G, Oris M (2008). Effects of Inheritance and Environment on the Heights of Brothers in Nineteenth-Century Belgium. Hum Nat (Hawthorne, NY).

[19] Barker DJ (2007). The origins of the developmental origins theory. J Intern Med.

[20] Barker DJ, Osmond C, Kajantie E, Eriksson JG (2009). Growth and chronic disease: findings in the Helsinki birth cohort. Ann Hum Biol.

[21] Law C, Barker D, Osmond C, Fall C, Simmonds S (1992). Early growth and abdominal fatness in adult life. J Epidemiol Community Health.

[22] Godfrey KM, Barker DJ (2000). Fetal nutrition and adult disease. Am J Clin Nutr.

[23] Xu H, Li L, Zhang Z, Liu J (2016). Is natural experiment a cure? Re-examining the long-term health effects of China's 1959–1961 famine. Soc Sci Med.

[24] Dercon S, Porter C (2014). Live aid revisited: long-term impacts of the 1984 Ethiopian famine on children. J Eur Econ Assoc.

[25] Ravelli AC, Van Der Meulen JH, Osmond C, Barker DJ, Bleker OP (1999). Obesity at the age of 50 y in men and women exposed to famine prenatally. Am J Clin Nutr.

[26] Ravelli G-P, Stein ZA, Susser MW (1976). Obesity in young men after famine exposure in utero and early infancy. N Engl J Med.

[27] van Abeelen AF, Elias SG, Roseboom TJ, Bossuyt PM, van der Schouw YT, Grobbee DE, et al. Postnatal acute famine and risk of overweight: the dutch hungerwinter study. Int J Pediatr. 2012;2012. 10.1155/2012/936509 PMC3352614 22611413

[28] Stein AD, Kahn HS, Rundle A, Zybert PA (2007). Van der pal–de Bruin K, Lumey L. anthropometric measures in middle age after exposure to famine during gestation: evidence from the Dutch famine. Am J Clin Nutr.

[29] Portrait FRM, van Wingerden TF, Deeg DJH (2017). Early life undernutrition and adult height: The Dutch famine of 1944–45. Econ Hum Biol.

[30] Chang X, Song P, Wang M, An L (2018). The risks of overweight, obesity and abdominal obesity in middle age after exposure to famine in early life: evidence from the China’s 1959–1961 famine. J Nutr Health Aging.

[31] Liu D, Yu DM, Zhao LY, Fang HY, Zhang J, Wang JZ, et al. Exposure to Famine During Early Life and Abdominal Obesity in Adulthood: Findings from the Great Chinese Famine During 1959(−)1961. Nutrients. 2019;11(4). 10.3390/nu11040903 PMC6521085 31013668

[32] Meng RR, Si JH, Lyu J, Guo Y, Bian Z, Yu CQ (2016). Association between famine exposure during early life and BMI in adulthood. Zhonghua Liu Xing Bing Xue Za Zhi.

[33] Wang Y, Wang X, Kong Y, Zhang JH, Zeng Q (2010). The great Chinese famine leads to shorter and overweight females in Chongqing Chinese population after 50 years. Obesity (Silver Spring).

[34] Arage G, Belachew T, Hassen H, Abera M, Abdulhay F, Abdulahi M, et al. Effects of prenatal exposure to the 1983-1985 Ethiopian great famine on metabolic syndrome in adults: a historical cohort study. Br J Nutr. 2020:1–27. 10.1017/S0007114520002123 32517836

[35] Riley L, Guthold R, Cowan M, Savin S, Bhatti L, Armstrong T (2016). The World Health Organization STEPwise approach to noncommunicable disease risk-factor surveillance: methods, challenges, and opportunities. Am J Public Health.

[36] Sinaga M, Worku M, Yemane T, Tegene E, Wakayo T, Girma T (2018). Optimal cut-off for obesity and markers of metabolic syndrome for Ethiopian adults. Nutr J.

[37] Vyas S, Kumaranayake L (2006). Constructing socio-economic status indices: how to use principal components analysis. Health Policy Plan.

[38] Humeniuk R (2008). Validation of the alcohol, smoking and substance involvement screening test (ASSIST). Addiction.

[39] Hagströmer M, Oja P, Sjöström M (2006). The International Physical Activity Questionnaire (IPAQ): a study of concurrent and construct validity. Public Health Nutr.

[40] Aragie T, Genanu S (2017). Level and Determinants of Food Security in North Wollo Zone (Amhara Region—Ethiopia). J Food Secur.

[41] Rodríguez MM (2002). Validation of a semi-quantitative food-frequency questionnaire for use among adults in Guatemala. Public Health Nutr.

[42] Huang C, Li Z, Wang M, Martorell R (2010). Early life exposure to the 1959–1961 Chinese famine has long-term health consequences. J Nutr.

[43] Meng X, Qian N. The long term consequences of famine on survivors: evidence from a unique natural experiment using China's great famine: National Bureau of Economic Research; 2009. Report No.: 0898–2937.

[44] Kuh D, Shlomo YB (2004). A life course approach to chronic disease epidemiology: Oxford University press.

[45] Powell GF, Brasel J, Blizzard R (1967). Emotional deprivation and growth retardation simulating idiopathic hypopituitarism: clinical evaluation of the syndrome. N Engl J Med.

[46] Bogin B, Hermanussen M, Scheffler C (2018). As tall as my peers–similarity in body height between migrants and hosts. Anthropol Anz.

[47] Tanjung C, Prawitasari T, Sjarif DR (2020). Comments on “stunting is not a synonym of malnutrition”. Eur J Clin Nutr.

[48] Hult M, Tornhammar P, Ueda P, Chima C, Bonamy A-KE, Ozumba B (2010). Hypertension, diabetes and overweight: looming legacies of the Biafran famine. PLoS One.

[49] Koupil I, Shestov DB, Sparén P, Plavinskaja S, Parfenova N, Vågerö D (2007). Blood pressure, hypertension and mortality from circulatory disease in men and women who survived the siege of Leningrad. Eur J Epidemiol.

[50] Stanner SA, Bulmer K, Andres C, Lantseva OE, Borodina V, Poteen V (1997). Does malnutrition in utero determine diabetes and coronary heart disease in adulthood? Results from the Leningrad siege study, a cross sectional study. Bmj..

[51] Desai M, Beall M, Ross MG (2013). Developmental origins of obesity: programmed adipogenesis. Curr Diab Rep.

[52] Ong KK (2006). Size at birth, post-natal growth and risk of obesity. Horm Res Paediatr.

[53] Monteiro POA, Victora CG (2005). Rapid growth in infancy and childhood and obesity in later life–a systematic review. Obes Rev.

[54] Eshetie T, Hussien K, Teshome T, Mekonnen A (2018). Meat production, consumption and marketing tradeoffs and potentials in Ethiopia and its effect on GDP growth: a review. J Nutr Health Food Eng.

[55] Weisell RC (2002). Body mass index as an indicator of obesity. Asia Pac J Clin Nutr.

[56] Woo J, Leung J, Wong S (2010). Impact of childhood experience of famine on late life health. J Nutr Health Aging.

[57] Zheng X, Wang Y, Ren W, Luo R, Zhang S, Zhang JH, Zeng Q (2012). Risk of metabolic syndrome in adults exposed to the great Chinese famine during the fetal life and early childhood. Eur J Clin Nutr.

[58] Zhou J, Sheng J, Fan Y, Zhu X, Tao Q, Liu K, Hu C, Liang R, Yang L, Tao F, Wang S (2019). The effect of Chinese famine exposure in early life on dietary patterns and chronic diseases of adults. Public Health Nutr.

[59] Myers JH (1978). Sex Ratio Adjustment Under Food Stress: Maximization of Quality or Numbers of Offspring?. Am Nat.

[60] Hadley C, Lindstrom D, Tessema F, Belachew T (2008). Gender bias in the food insecurity experience of Ethiopian adolescents. Soc Sci Med.

