# Supplementary material for: Impact of early life famine exposure on adulthood anthropometry among survivors of the 1983–1985 Ethiopian Great famine: a historical cohort study
Source: BMC Public Health. 2021 Jan 7;21:94. doi: 10.1186/s12889-020-09982-x (PMC7792120; doi:10.1186/s12889-020-09982-x)
Supplement: Supplementary file 1 — Additional file 1: Table 1: Window of exposure to the 1983–85 Ethiopian Great Famine birth cohorts, Raya Kobo District, Northeast Ethiopia, 2019. [file 12889_2020_9982_MOESM1_ESM.docx]

**Table 1: Window of exposure to the 1983-85 Ethiopian Great Famine birth cohorts, Raya Kobo District, Northeast Ethiopia, 2019.**

| **Birth year of participants (dd/mm/yyy)** | **Exposure to the famine August 1983, August 1985** | **Age of exposure to famine** | **Age at recruitment** |
| --- | --- | --- | --- |
| 08/August/1983-30/August /1985 | Prenatal exposed | born or conceived during the famine | 34-36 |
| 08/September/1986 30/August/1987 | Transition (Washout period) | One years after the famine | 33 |
| 08/September/1981-30/August/1983 | Postnatal exposed | 0 – 2 years old | 37-38 |
| 08/September/1976- 30/August/1980 | Mid-childhood exposed | 3 – 9 years old | 39-43 |
| 08/September1964-30/August/1975 | Adolescent exposed | 10-19 years old | 44-55 |
| 08/September/1987-08/October/1988 | No exposure (reference group) | Two years old after the famine | 30-32 |
| 08/September/1964 -30/August/1985 ^a^ | Early life exposed ^a^ | Prenatal + postnatal + adolescent exposed | 34 - 55^a^ |

^a^ Exclude mid-child hood exposed
